# Evidence for an ice shelf covering the central Arctic Ocean during the penultimate glaciation

**DOI:** 10.1038/ncomms10365

**Published:** 2016-01-18

**Authors:** Martin Jakobsson, Johan Nilsson, Leif Anderson, Jan Backman, Göran Björk, Thomas M. Cronin, Nina Kirchner, Andrey Koshurnikov, Larry Mayer, Riko Noormets, Matthew O'Regan, Christian Stranne, Roman Ananiev, Natalia Barrientos Macho, Denis Cherniykh, Helen Coxall, Björn Eriksson, Tom Flodén, Laura Gemery, Örjan Gustafsson, Kevin Jerram, Carina Johansson, Alexey Khortov, Rezwan Mohammad, Igor Semiletov

**Affiliations:** 1 Department of Geological Sciences, Stockholm University, Stockholm 106 91, Sweden; 2 Bolin Centre for Climate Research, Stockholm University, Stockholm 106 91, Sweden; 3 UNIS - The University Centre in Svalbard, Longyearbyen N-9171, Svalbard; 4 Department of Meteorology, Stockholm University, Stockholm 106 91, Sweden; 5 Department of Marine Sciences, University of Gothenburg, Gothenburg 405 30, Sweden; 6 US Geological Survey Reston, 12201 Sunrise Valley Drive, Reston, Virginia 20192, USA; 7 Department of Physical Geography, Stockholm University, Stockholm 106 91, Sweden; 8 National Research Tomsk Polytechnic University, Tomsk 634050, Russia; 9 Department of Geocryology, Moscow State University, Moscow 119991, Russia; 10 Center for Coastal and Ocean Mapping, University of New Hampshire, 24 Colovos Road, Durham, New Hampshire 03824, USA; 11 Russian Academy of Sciences, Pacific Oceanological Institute, 43 Baltiiskaya Street, Vladivostok 690041, Russia; 12 Department of Environmental Science and Analytical Chemistry, Stockholm University, Stockholm 106 91, Sweden

## Abstract

The hypothesis of a km-thick ice shelf covering the entire Arctic Ocean during peak glacial conditions was proposed nearly half a century ago. Floating ice shelves preserve few direct traces after their disappearance, making reconstructions difficult. Seafloor imprints of ice shelves should, however, exist where ice grounded along their flow paths. Here we present new evidence of ice-shelf groundings on bathymetric highs in the central Arctic Ocean, resurrecting the concept of an ice shelf extending over the entire central Arctic Ocean during at least one previous ice age. New and previously mapped glacial landforms together reveal flow of a spatially coherent, in some regions >1-km thick, central Arctic Ocean ice shelf dated to marine isotope stage 6 (∼140 ka). Bathymetric highs were likely critical in the ice-shelf development by acting as pinning points where stabilizing ice rises formed, thereby providing sufficient back stress to allow ice shelf thickening.

## Abstract (web summary)

The development of pan-Arctic Ocean ice shelves during peak glacials was proposed in the 1970s, an idea that has been disputed due to lack of evidence. Here, the authors present geophysical mapping data supporting the presence of such an ice shelf during the peak of the penultimate glaciation ∼140–160 ka.

## Graphical abstract

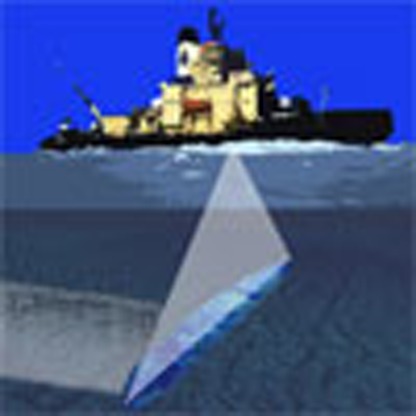

Ice conditions in the Arctic Ocean during glacial maxima have been much debated, with hypotheses formulated long before direct observational data existed. In 1888, Sir William Thomson speculated about extensive and thick floating ice, and elaborated on possible effects of isolating Arctic Ocean water masses from the remaining World Ocean^1^. Nearly a century later, speculations ranged from a sea-ice-free Arctic Ocean during glacial maxima^2^ to one where an extensive and thick ice shelf persisted^3^,^4^,^5^. In the mid-1960s, it was proposed that large portions of the Barents Sea had been covered by a marine ice sheet during the last glacial maximum (LGM)^6^. In 1970, Mercer pointed out striking similarities between the glacial-age Arctic Ocean and today's West Antarctic ice sheet, where extensive ice shelves exist, and he stressed that the idea of an Arctic Ocean filled by thick ice was glaciologically sound and should be taken seriously^3^. Additional support for Arctic Ocean ice caps with huge floating parts in the form of ice shelves was shortly thereafter provided by Broecker^4^ and Hughes *et al*.^5^, who proposed a thick, floating and dynamic ice shelf during the LGM on the basis that such an ice shelf may have been necessary to stabilize the inherently unstable marine ice sheets located on the Arctic's continental margins (Fig. 1a). The concept of a dynamic ice shelf was developed further in some Arctic Ocean ice sheet reconstructions for the LGM^7^,^8^.

When the ice-shelf theory was developed in the 1970s and 1980s, the climatic implications of a huge floating Arctic Ocean ice shelf and extensive sea ice were addressed^8^ along with its potential effect on the global ocean δ^18^O record measured in benthic foraminifera^4^,^9^,^10^. It was during this period specifically noted that the amplitude of δ^18^O variations in benthic foraminifera predicts more ice volume than available sea-level records indicate^11^, a discrepancy that may be explained if ^16^O is stored in a huge floating ice shelf that, once melted, has only a minor effect on sea level^4^,^9^,^10^. Lack of direct evidence, however, destined the notion of an Arctic Ocean-wide ice shelf eventually to relative obscurity, and the discrepancy between ice volume inferred from δ^18^O and geological sea-level records was in addition suggested to be caused mainly by massive Antarctic ice shelves^12^.

Seafloor mapping of the Yermak Plateau off northern Svalbard provided the first evidence of thick glacial ice grounding in the Arctic Ocean^13^ (Fig. 1b). This was followed by the discovery of seafloor ice erosion at depths approaching 1,000 m on the Lomonosov Ridge (LR) and the Chukchi Borderland^14^,^15^. These results, together with dating of sediment cores from the eroded areas, pointed to an ice shelf constrained to the Amerasian Basin during marine isotope stage (MIS) 6 (∼140–160 ka)^16^ (Fig. 1b). There were two main reasons for limiting this ice shelf to the Amerasian Basin: (1) previous mapping of <1,000 m deep sectors of the LR between 84°30′ N and the Siberian margin had not revealed ice grounding^17^, and (2) the ice erosion mapped on the central LR was assumed to have been caused by armadas of icebergs rather than a coherent ice shelf^16^,^18^.

Here we present new multibeam bathymetry and sub-bottom profiles documenting ice scours and other glacial landforms extending across the central Arctic Ocean that compel us to assess the concept of a coherent ∼1-km thick ice shelf extending over the entire Arctic Ocean. Our new observations from the LR off the Siberian margin, the Arlis Plateau and the continental slope north off Herald Canyon (Fig. 1b), merged with published observations, require an Arctic ice shelf close to the ‘maximum ice' scenario, in terms of thickness, area and flow pattern, as hypothesized by Hughes *et al*.^5^. The new seafloor mapping data were collected during the SWERUS-C3 (Swedish–Russian–US Arctic Ocean Investigation of climate–cryosphere–carbon interactions) expedition in 2014.

## Results

### Geophysical mapping

Two sets of highly parallel streamlined submarine landforms cross the southern LR crest at about 81° N 143° E (Fig. 2). These features consist of approximately 10–15 high ridges that are spaced between 400 and 800 m apart. Morphologically, the mapped landforms closely match mega-scale lineations that are widely found in formerly glaciated continental margins where they are interpreted to signify fast-flowing ice streams^19^. The LR crest is in the area of 81° N 143° E and is shaped by ice grounding with a gently sloping stoss side towards the Makarov Basin and a steep lee side facing the Amundsen Basin (Fig. 2a,b). The general ice-flow direction is diagonally across the LR towards northwest, from Makarov Basin to Amundsen Basin. Lineations extend as deep as 1,280 m below present sea level on the stoss side. The flattened ridge crest contains small arcuate ridges with a relief of about 6 m and their pointed edges facing southward, towards the youngest ice-flow direction across the ridge. There are faint indications of what may be grounding zone wedges on the flat ridge crest (Fig. 2b). The flat-topped nature of the ridge crest is caused at least in part by ice grounding, as evident by an unconformity visible in sub-bottom profiles (Fig. 3). Emergence and subsidence could generate a similar flat-topped appearance of the ridge crest, but only on much longer time scales^20^. Further north at about 85° N and 153° E, the LR shows a more accentuated flat-topped ridge crest formed by ice grounding between about 1,000 and 700 m water depth (Figs 2c and 4). Also in this area, the ridge slope facing the Makarov Basin is gentler than towards the Amundsen Basin, suggesting a stoss and lee side with respect to the ice-shelf flow. Remarkably consistent parallel lineations extend diagonally across the ridge towards west–northwest. At about 84°15′ N there is a section of the LR crest at around 890 m present water depth where the ice shelf apparently did not ground and where giant pockmarks dominate the seafloor morphology (Supplementary Figs 1 and 2).

The Arlis Plateau (Fig. 2d) was also mapped during SWERUS-C3 to complement previously mapped glacial lineations extending to ∼1,200 m depth, interpreted to represent grounding of an ice shelf extending from the East Siberian margin^21^. We mapped the intersection between two distinct sets of lineations on the Arlis Plateau crest (Fig. 2d). Superposition demonstrates that lineations having directions towards east–northeast, rather than northeast, are older (white arrows in Fig. 2d).

In addition, the seabed of the Chukchi Borderland is heavily affected by ice grounding^14^,^22^. Mapped patterns of glacial landforms have been interpreted to show a large ice rise belonging to the MIS 6 Amerasian ice shelf and marine outlet glaciers emanating from hypothesized East Siberian ice sheets^21^,^22^,^23^. Mapping of the slope west of the Chukchi Borderland north of Herald Canyon during the SWERUS-C3 expedition shows distinct sets of ridges diagonal to the dip of the slope in water depths from about 390 to >600 m (Fig. 2e). Morphologically, these ridges resemble recessional moraines perpendicular to the past ice flow^24^. Their direction suggests ice flow from the Chukchi Sea margin where the Herald Canyon ends. Similar ridges have been previously mapped further downslope at about 700 m water depth^21^.

### Dating of ice grounding

Sediments deposited atop the ice-grounded surfaces on bathymetric highs in the central Arctic Ocean have been dated using several methods: radiocarbon dating of MIS 3-1 (refs 25, 26), astronomical tuning of sediment physical and chemical properties^27^,^28^, calcareous nannofossil biostratigraphy of MIS 5 (ref. 29), inter-core correlation based on dinoflagellate cysts^30^, and benthic and planktic foraminifera^31^,^32^.

New data for SWERUS-C3 sediment cores from the ice-grounded areas are shown in Fig. 5 and their locations are shown in Fig. 2 and Supplementary Fig. 3. Acoustically laminated sediments overlie the eroded surfaces. On the central LR, core SWERUS-L2-32-GC2 (32-GC2 on map, Fig. 2c, and on sub-bottom profile in Supplementary Fig. 3) can be accurately correlated to core 96/12-1PC, which has a well-constrained age model back to MIS 6 (ref. 28; Fig. 5). The correlations are based on physical property variations, that is, magnetics susceptibility and bulk density, captured in high-resolution multi-sensor core-logging measurements. The developed correlation indicates that the 2.5 m gravity core recovered an undisturbed sedimentary section back to MIS 5.5, constraining the ice scouring event at this site to MIS 6 or older. The correlation, which places MIS 5 between 1.5 and 2.35 m.b.s.f. (metre below seafloor), is supported by rare occurrences of the calcareous nannofossil *Emiliania huxleyi* in three samples at 1.69–1.72 m.b.s.f.

On the Southern LR, the 4.66-m long core SWERUS-L2-29-GC1 (29-GC1 on map, Fig. 2a,b, and on sub-bottom profile in Supplementary Fig. 3) sampled acoustically stratified sediments deposited on top of the ice-scoured surface. This core was collected in 824 m water depth. Physical properties from this core are correlated to neighbouring core collected by the *Polarstern* in 1995 (PS2757-8) (Fig. 5). This core was recovered from a water depth of 1,241 m, and is from just below the maximum ice-grounding surface on this portion of the LR (Fig. 2a). The entire recovered sedimentary sequence in SWERUS-L2-29-GC1 is mirrored in the deeper lying core, indicating that the erosional surface lies at the base of this core. Calcareous nannofossils indicate a Holocene age in the uppermost 5 cm of this core, with rare occurrences of *E. huxleyi*, *C. leptoporus* and *G. muellerae*. No age diagnostic microfossils were observed below 0.05 m.b.s.f., although one potential observation of *E. huxleyi* was made in the sample at 3.81 m.b.s.f. The base of PS-2757 has previously been assigned an MIS 6 age through correlation of organic geochemical parameters and magnetic susceptibility measurements to better-dated records on the Laptev and Barents Sea slope^30^. Although this age assignment remains speculative, radiocarbon dating of the upper 10 cm of PS2757-8, and dinocyst abundance and assemblage data, firmly place the base of the Holocene at 0.6 m.b.s.f., suggesting sedimentation rates between 5 and 7 cm per ka (ref. 30). Extrapolating these sedimentation rates downcore suggests that the base of PS2757-8 is younger than 200 ka. Conclusively, available data show that the ice-scoured surface is older than the LGM, and likely occurred during MIS 6.

On the Arlis Plateau, core SWERUS-L2-13-PC1 (13-PC1 on map, Fig. 2d, and on sub-bottom profile in Supplementary Fig. 3) recovered 6.14 m of sediment at a water depth of 1,119 m where the seafloor has been subjected to ice grounding. A dark brown layer between 2.64 and 2.96 m.b.s.f. had rare nannofossils in three samples. The 2.86 m.b.s.f. sample yielded rare *E. huxleyi* and *Gephyrocapsa* spp., suggesting a MIS 5 age.

## Discussion

**Figure 1:**
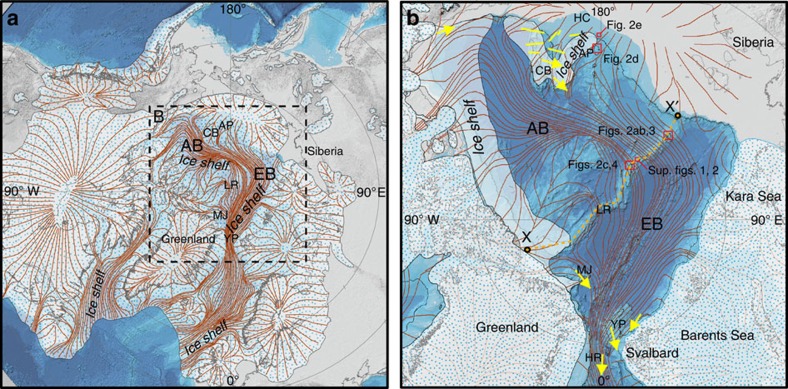
Ice-sheet reconstructions during glacial conditions involving ice shelves in the Arctic Ocean. (**a**) LGM ice-sheet reconstruction by Hughes *et al*.^5^ with an ice shelf that covers the entire Arctic Ocean and extends into the North Atlantic. Brown lines represent inferred ice-sheet flow. The modern coastline is used as a reference. (**b**) The limited ice shelf proposed by Jakobsson *et al*.^16^ is shown as white semi-transparent area. The extent of the MIS 6 (Late Saalian) Barents–Kara Sea ice sheet^49^ is shown as white semi-transparent blue dotted area. The North American ice sheet (late Wisconsinan^50^, also blue dotted area, is assumed to have been similar to the Illinoian ice sheet (MIS 6). Yellow arrows represent previously published evidence of ice-shelf grounding and interpreted flow direction^16^,^22^,^34^,^51^. Flow lines from Hughes *et al*.^5^ are shown also in **b** for comparison with ice-sheet flow inferred from mapped landforms. The orange dashed line X to X' marks the bathymetric profile in Fig. 7a. The black contour line in **b** represent the present day 1,000 m isobaths (Sup. Figs 1 and 2 refer to Supplementary Figs 1 and 2). The modern coastline is used as a reference. AB, Amerasian Basin; AP, Arlis Plateau; CB, Chukchi Borderland; EB, Eurasian Basin; HC, Herald Canyon; HR, Hovgaard Ridge; LR, Lomonosov Ridge; MJ, Morris Jesup Rise; YP, Yermak Plateau.

**Figure 2:**
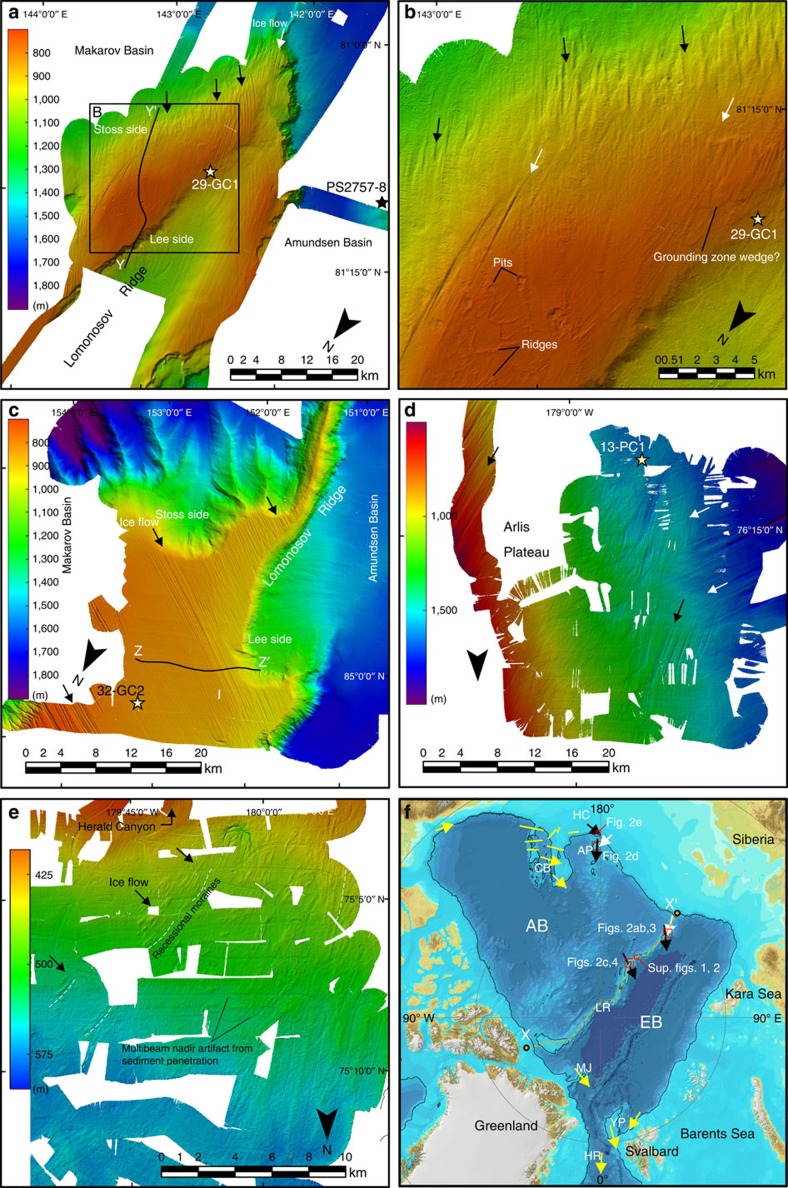
Multibeam bathymetry of submarine glacial landforms mapped during the SWERUS-C3 expedition. The SWERUS (Swedish–Russian–US Arctic Ocean Investigation of Climate–Cryosphere–Carbon Interactions) exhibition, 2014 data from bathymetric highs are interpreted to signify ice-shelf grounding. (**a**–**c**) Lomonosov Ridge (**b** is a detail of **a**), (**d**) Arlis Plateau and (**e**) the slope north of Herald Canyon. The locations of all inset maps are shown in Fig. 1b as well as in **f**, where the present day bathymetry from the International Bathymetric Chart of the Arctic Ocean (IBCAO) is shown^52^. The 1,000 m isobaths is shown as bathymetric reference in black in **f**. Yellow arrows in **f** represent previously published evidence of ice-shelf grounding and interpreted flow direction^16^,^22^,^34^,^51^. Chirp sonar profiles between Y–Y' and Z–Z' are shown in Figs 3 and 4, respectively. The location of SWERUS-C3 cores used to date the ice-shelf grounding are marked with yellow stars and the stratigraphically correlated core PS2757-8 (ref. 30) is shown with a black star. See caption of Fig. 1 for used abbreviations undersea features.

**Figure 3:**
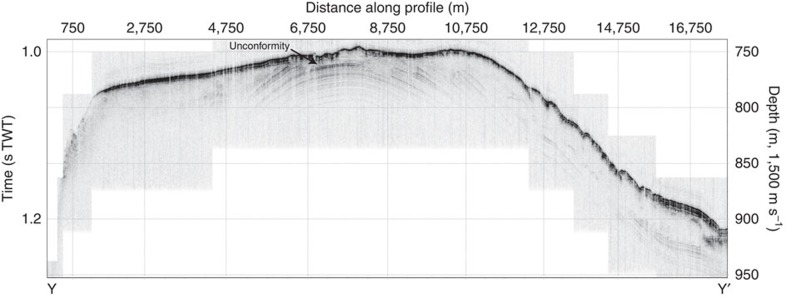
Chirp sonar sub-bottom profile from the southern Lomonosov Ridge. The location of the profile between Y and Y' is shown in Fig. 2a. The erosional unconformity, formed by ice grounding, is indicated in the profile.

**Figure 4:**
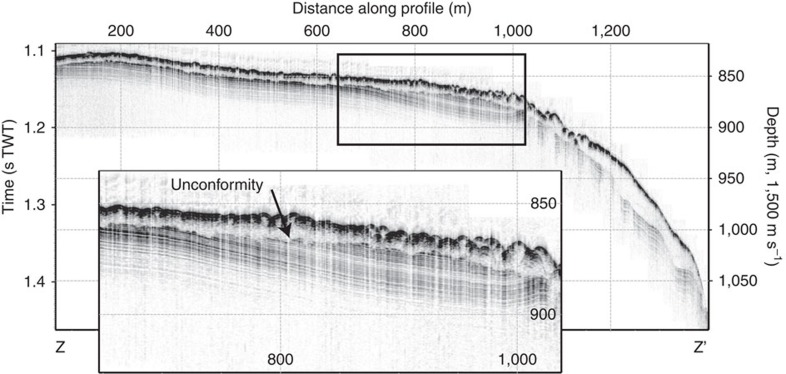
Chirp sonar sub-bottom profile from the central Lomonosov Ridge. The location of the profile between Z and Z' is shown in Fig. 2c. The erosional unconformity, formed by ice grounding, is indicated in the profile.

Taken together, the new results suggest that an ice shelf existed during MIS 6 that was thicker and covered substantially more of the Arctic Ocean than previously suggested (Fig. 1b)^16^. A minimum scenario suggests an ice shelf during MIS 6 that covered most of, if not the entire, Amerasian Basin. Bathymetric highs generally shallower than ∼1,000 m present water depth (at southern LR as deep as 1,280 m) acted as stabilizing pinning points through the formation of ice rises/rumples. Not all <1,000 m parts of the LR acted as pinning points, since a few sections are untouched by the ice shelf, but these may be explained by an uneven ice thickness (Fig. 6). It may at first seem reasonable to limit this ice shelf to the Amerasian Basin and the LR, however, previously mapped lineations on the Yermak Plateau^16^,^33^ fit well with the hypothesized flow pattern suggested by Hughes *et al*.^5^. Suggested causes for the lineations on the Yermak Plateau include grounding of a larger ice-shelf fragment originating from the Amerasian Basin, an armada of large icebergs, or an ice-sheet component extending northward into the Arctic Ocean from the Barents Sea ice sheet^16^,^33^. The morphological similarity between the lineations previously mapped on the Yermak Plateau and those mapped on the LR during SWERUS-C3, together with their flow direction, suggest that they all originate from grounding of an Arctic Ocean-wide ice shelf, similar to the suggestion of Hughes *et al*.^5^. If several smaller ice shelves at different times during the MIS 6 glaciation instead were responsible for the mapped glacial landforms, the spatially coherent pattern over the central Arctic Ocean is difficult to explain. The recently discovered deep scours reaching water depths >1,200 m on the Hovgaard Ridge, located south of the Fram Strait, are interpreted to indicate a massive outflow of large deep-drafting icebergs from the Arctic Ocean^34^. Additional detailed mapping of this ridge and other bathymetric highs south of the Fram Strait is required to rule out the possibility that the Arctic Ocean ice shelf did not extend into the Norwegian–Greenland seas as suggested by Hughes *et al*.^5^ With no data at hand, we assume that the MIS 6 ice shelf was limited to the central Arctic Ocean.

The age(s) of deep ice grounding in the central Arctic Ocean have been discussed since the first evidence was mapped on the central LR and sediment cores from this area were dated^14^,^35^. The assigned MIS 6 age stems from the fact that there is a rather systematic drape of sediment beginning from MIS 5.5 atop the mapped glacial landforms and glacially eroded surfaces. However, it should be noted that Chukchi Borderland generally has a more complicated sediment stratigraphy with indications of additional glacial erosional events younger than MIS 6; the most recent is suggested to have occurred during MIS 4 (ref. 36). We cannot at present exclude the possibility of occurrences of thinner ice shelves younger than MIS 6 over large areas of the central Arctic Ocean that did not reach bathymetric highs as deep as ∼1,000 m. Neither can we rule out that large ice shelves existed during older glacials than MIS 6 (for example, MIS 8 or 12) since evidence in the form of glacial landforms on bathymetric highs may have been erased by the most recent event during MIS 6.

While the morphological evidence suggests an Arctic Ocean-wide ice shelf at MIS 6, is such a feature oceanographically possible? The answer to this question lies in the details of the oceanographic conditions at the time. The present influx of >3 Sv (1 Sv=10^6^ m^3^ s^-1^) of warm (>0 °C) Atlantic water between about 200 and 600 m, would strongly impact negatively on ice-shelf development^16^. It has been suggested that Atlantic water was forced deeper in the central Arctic Ocean during glacials^16^,^37^, thus limiting Atlantic flow across the LR into the Amerasian Basin. Oceanographic conditions in the Amerasian Basin during MIS 6 may be characterized as an extreme version of conditions in present-day cavities below Antarctic ice shelves^38^. However, as our mapping results from the LR suggest that the MIS 6 ice shelf extended into the Amundsen Basin, it likely was in contact with warm Atlantic water. This would result in a circulation where water warmer than the freezing point of ice melts the underside of the ice shelf to produce cool fresher water that strives to rise towards the surface. Could this kind of circulation have reached all the way across the LR and into the Amerasian basin due to that warmer Atlantic water flowed over the ridge? Wide bathymetric passages in the ridge below the grounded ice shelf (Fig. 6) may have formed conduits for warm water causing melting also in the Amerasian Basin. This type of circulation is known from Antarctica to have large horizontal variability, and uneven melting might have formed an irregular underside of the ice shelf explaining the uneven grounding depth indicated by seafloor mapping data (Fig. 6).

Based on previous work^16^, we formulate a conceptual two-layer ocean model on the flow underneath the ice shelf, that is, the ice cavity (Fig. 6). The aim is to obtain a simple model of the melt rate in a large ice cavity with a highly restricted exchange flow. For the sake of simplicity, we assume a steady state and ignore spatial variations below the ice shelf, having an essentially constant thickness over the Amerasian Basin. The water in the cavity that is in contact with the ice shelf is assumed to be at freezing point, which decreases with increasing depth (pressure) of the base of the ice shelf. Continuity of volume is given by


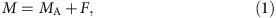


where *M* is volume outflow from the ice cavity, *M*_A_ the oceanic inflow, and *F* the net freshwater supply due to ice melt or growth in the cavity. The remaining components of this model, based on conservation of salt and heat, are described in the Methods. For a reasonable choice of parameters, the resulting volume flow *M* is on the order of 1 Sv and the oceanic heat flux to the base of the shelf is on the order of 1 W m^−2^, corresponding to an ice melt of about 0.1 m per year. Thus, this highly simplified model suggest that if the turbulent mixing intensity in the cavity is weak, as is reasonable to assume, then the basal melting of the shelf would likely be less than snow accumulation on the top of the ice shelf. In turn, this indicates that the ocean-induced basal melting was weak enough to allow for a 1-km thick ice shelf over the Arctic Ocean as indicated by geophysical mapping data from bathymetric highs.

The geometry of the nearly landlocked Arctic Ocean likely played a major role for the formation of the MIS 6 ice shelf^39^. The Ross Ice Shelf, West Antarctica, fills its embayment even in today's ‘warm' climate. Ice flux over its grounding line and calving flux are approximately in balance (∼150 Gt per year), while surface mass balance outweighs underside melt by ∼4 Gt per year, implying an overall positive mass balance^40^. Major ice streams in the Arctic drained the Laurentide Ice and Barents–Kara ice sheets and fed the MIS 6 ice shelf (Fig. 1). Stabilized by perennial sea ice during inception, and by grounding on bathymetric highs, the ice shelf would have successively filled the entire central Arctic Ocean embayment. MIS 6 climate modelling studies indicate surface mass accumulation rates on the order 0.15–0.2 m per year for an ice shelf covering the entire Arctic Ocean^41^, further supporting that a positive mass balance could be sustained if basal melt is less than 0.1 m per year. Furthermore, simulations of the MIS 6 glacial maximum, ∼140 ka, using a coupled Atmosphere–Ocean–Sea-Ice–Land model yield air surface temperatures over the Arctic Ocean that were 12–16 °C colder than pre-industrial temperatures, leading to perennial sea-ice cover reaching >10 m thickness over the entire Arctic Ocean^42^. These simulations, although made without an ice shelf, can be used in the following physical consideration: over the long timescales considered here, the ice-shelf temperature distribution may be approximated to be constant at a certain time and certain ice-shelf thickness. As a consequence, the vertical heat flux is constant through the ice shelf, which from the heat conduction law implies a linear temperature profile^43^. At the base of the ice shelf, the temperature equals the local freezing point of the sea water, which is about −2.5 °C for a 1,000-m thick ice shelf. Taking the simulated surface temperatures^42^ and the physically constrained basal temperature, the heat conduction law gives a vertical heat flux of about 0.1 W m^−2^ through the MIS 6 ice shelf. The simple oceanographic model presented here suggests that the heat flux from the ocean to the ice is an order of magnitude greater. Thus, melting, rather than accretion, is expected at the base of the MIS 6 ice shelf.

**Figure 5:**
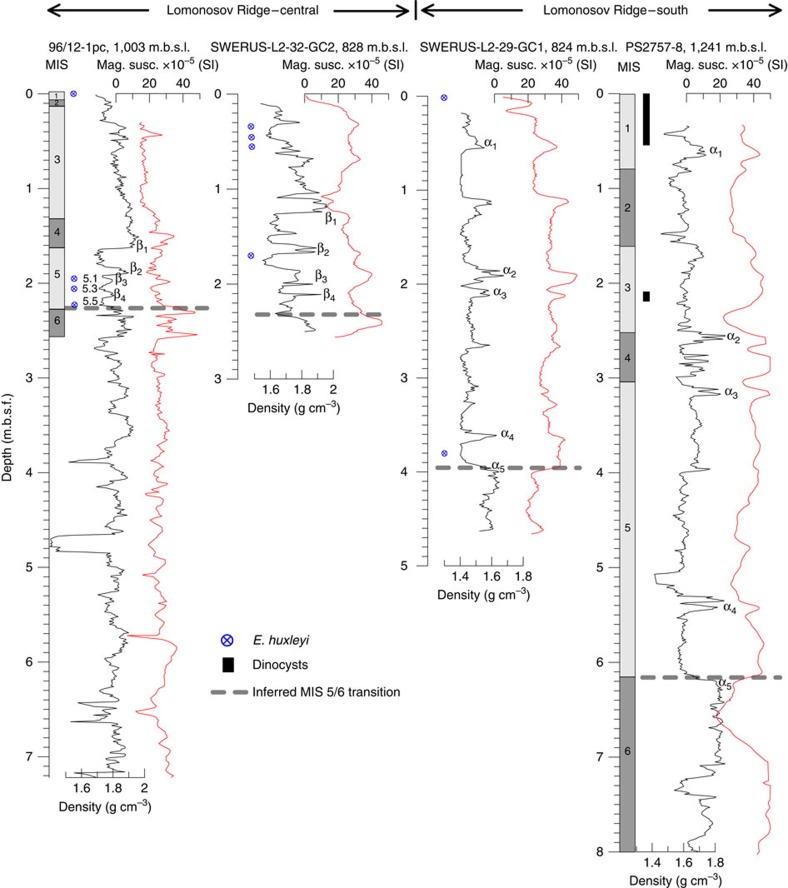
Stratigraphic correlations between cores from SWERUS-C3 and previously collected and dated sediments cores from the Lomonosov Ridge. Bulk density and magnetic susceptibility of core 96/12-1pc from the central Lomonosov Ridge is shown with previously inferred bulk density stratigraphic tie points β_1_–β_4_ (ref. 35) linking it to nearby SWERUS-L2-32-GC2 (short name 32-GC2 on map in Fig. 2c). Bulk density and magnetic susceptibility records from SWERUS-L2-29-GC1 (short name 29-GC1 shown on map in Fig. 2a) correlated to core PS2757-8, both from the southern Lomonosov Ridge off the Siberian continental margin. The age models for 96/12-1pc (ref. 28) and PS2757-8 (ref. 30) are shown by the bar to the left with inferred MIS 1-6.

**Figure 6:**
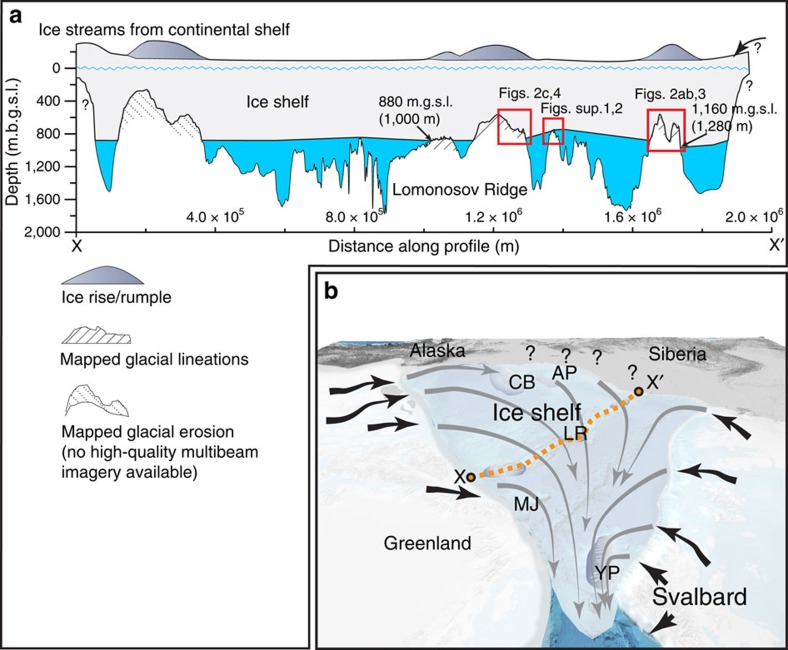
Conceptual sketches of ice shelf covering the entire central Arctic Ocean. (**a**) Bathymetric profile along the LR, from the Greenland continental margin (X) to the margin off the New Siberian Islands (X'). See profile location in **b** and Fig. 1b. (**b**) Sketch of an ice shelf covering the entire central Arctic Ocean with flow lines generalized from mapped glacial landforms. The locations of ice rises/rumples on bathymetric highs, inferred from mapped landforms, are shown with the darker grey shading (see legend in **a**). m.g.s.l. refers to depth in metres below glacial sea level, here defined as 120 m below present sea level. The inferred general flow lines of the floating ice shelf (grey) are tapered to illustrate that the shelf thickness would thin with the distance from feeding ice streams (black arrows) and grounding lines, except for where the shelf grounded on bathymetric highs to form local ice rises/rumples. A positive mass balance of the ice shelf would be sustained if accumulation rates through precipitation in the central Arctic Ocean are higher than basal melting and mass loss at the calving front (see model of ocean–ice–cavity interaction). The locations of undersea features where ice grounding and additional glacial landforms have been mapped are marked with abbreviations: AP, Arlis Plateau; CB, Chukchi Borderland; LR, Lomonosov Ridge; MJ, Morris Jesup Rise; YP, Yermak Plateau.

We propose that the area of the MIS 6 ice shelf roughly coincided with the central Arctic Ocean basin area, from the shelf break to the North Pole (Fig. 5b). This area is here calculated to about 4.25 × 10^6^ km^2^. The glacial landforms signifying ice-shelf grounding on the LR suggest that the ice shelf reached as deep as 1,280 m below present sea level where the ridge approaches the Siberian continental margin and 1,200 m on the Arlis Plateau. However, there are areas in the central Arctic Ocean where the ice shelf was thinner (Fig. 5b), and it was likely thicker near grounding lines. Assuming a sea level of ∼121 m lower than today for MIS 6 (ref. 44), we may approximate the average ice shelf draft to 1,000 m in the central Arctic Ocean basin. This implies an average thickness of 1,121 m using an ocean density of 1,028 kg m^−3^ and ice density of 917 kg m^−3^. Adopting the Archimedes principle, the net sea-level-rise effect from melting a floating ice mass is equivalent to the difference between density of sea water and that of the melted ice^45^. The Arctic Ocean ice shelf with an average thickness of 1,121 m and an aerial extent as outlined in Fig. 5b has a volume of ∼4.67 × 10^6^ km^3^, which if melted would approximately provide a sea-level rise of ∼0.34 m. This rough estimate is derived using a world ocean area compensated for a 121-m lower glacial sea level than at present and assuming an equal spread of the meltwater, which we recognize is a simplified view of the sea-level change. We note that the estimated volume of the MIS 6 Arctic Ocean ice shelf amounts to approximately seven times of the volume of all ice shelves on Earth today.

Shackleton and Opdyke^46^ showed in the early 1970s that benthic foraminifera from the equatorial Pacific recorded glacial/interglacial δ^18^O differences on the order of 1.7‰. In light of these results and subsequent δ^18^O paleoceanographic records derived from deep-sea sediment cores, the question of how much a thick Arctic Ocean ice shelf would affect the global ocean δ^18^O, while only having a minor effect on sea level, was addressed^4^,^9^,^10^. Mix^9^ used a δ^18^O value for shelf ice of −40±10‰ (SMOW) to estimate that a 700±300-m thick ice shelf covering the entire Arctic Ocean would increase the δ^18^O ocean value by 0.12±0.09‰ (SMOW). It follows that using a δ^18^O value for shelf ice of −40±10‰ (SMOW), and the estimated MIS 6 ice-shelf volume of 4.67 × 10^6^ km^3^, the MIS 6 δ^18^O ocean can be calculated to have increased by 0.14±0.03‰ (SMOW). This difference in calculated δ^18^O ocean compared with Mix^9^ is due to the larger volume of the MIS 6 ice shelf. Since the MIS 6 ice shelf only affects the eustatic sea level by ∼0.34 m, this should be taken into account when interpreting the global ocean δ^18^O value and its relation to global eustatic sea level of that time.

Finally, we emphasize that our results do not exclude the possibility that large ice shelves developed in the central Arctic Ocean during other glaciations than MIS 6. On the contrary, it seems likely that ice shelves were reoccurring components in most of the Quaternary glaciations considering the land-locked nature of the Arctic Ocean. Furthermore, the difficulty in precisely dating Arctic Ocean sediments leaves a high degree of uncertainty regarding the timing of several glacial landforms in general. However, the development of the large MIS 6 ice shelf may, in part, have been catalysed by the long duration of this glacial stage, where the eccentricity of Earth's orbit during the glacial maximum ∼140 ka caused particularly cold springs and summers^47^.

## Methods

### Geophysical mapping

The new seafloor mapping data presented in this work were collected during a two-leg 90-day long expedition in 2014 with Swedish *IB Oden* expedition within the Swedish–Russian–US Investigation of Climate, Cryosphere and Carbon interaction (SWERUS-C3) program. The expedition started/ended in Tromsö, Norway, July 5/October 3. Rotation between the two legs was carried out in Barrow, Alaska. The data presented in this work were collected during Leg 2 and outside the Russian Exclusive Economic Zone.

Bathymetric mapping was carried out using the Kongsberg EM 122 (12 kHz, 1° × 1°) multibeam echosounder hull mounted in *IB Oden*. This system has a Seatex Seapath 330 unit for integration of GPS navigation, heading and attitude. Sound velocity control was achieved through regular CTD (conductivity, temperature and depth) stations supplemented with XBT (expendable bathythermograph). All data were acquired using Kongsberg Seafloor Information System (SIS) and processed using a combination of the software Caris and Fledermaus-QPS. The processed data were gridded to a horizontal resolution ranging between 15 × 15 and 30 × 30 m. Seafloor morphology was interpreted in the three-dimensional environment of Fledermaus and maps were subsequently produced in the GIS software ArcMap. Sub-bottom profiles were collected using the Kongsberg SBP 120 3° × 3° chirp sonar integrated with the multibeam in *IB Oden*. The chirp sonar was operated continuously using a 2.5–7-kHz pulse. Any use of trade, firm or product names is for descriptive purposes only and does not imply endorsement by the US Government.

### A simple model of ocean–ice–cavity interaction

Following equation (1), the remaining components of our two-layer ocean model are as follows: conservation of salt can be written as


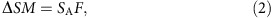


where Δ*S*≡*S*_A_−*S* here is the salinity difference between inflowing water with salinity *S*_A_ and the outflowing ice-shelf water with salinity *S*. The thermodynamic balance of the ice-shelf water layer, which governs the basal melt *F*, is given by


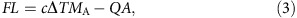


where *c* is the heat capacity of sea water, Δ*T*≡*T*_A_*−T*_f_ the temperature difference, *T*_f_ the freezing temperature at the hydrostatic pressure of the shelf ice base, *Q* the upward heat flow per unit area through the ice, *A* the shelf area and *L* the latent heat of freezing. By combining the thermodynamic relation with equation (1) it is possible to obtain


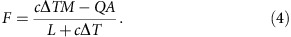


Combining this with the salinity equation yields


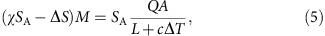


where we have introduced


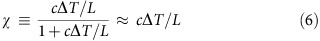


Here, *χS*_A_ represents a characteristic salinity difference; Δ*T* ∼ 2 °C, gives *χ* ∼ 0.03. We will here consider the limiting case where the conductive heat flux through the ice is small compared with the ocean heat flux, that is, assuming that *c*Δ*TM* >> *QA.* This is reasonable for thick shelf ice and with this limit equations (5) and (6) yield


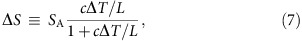


implying that the density difference is given by


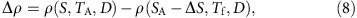


where *D* is the pressure at the ice-shelf base. Thus the salinity and density difference depends only on the salinity and temperature outside the cavity and the pressure at the shelf base. As Δ*ρ* is taken to be known, we can use the results of ref. 16 to compute the volume flow *M* and the upper-layer depth *H* in the cavity. We provide here a short summary of the physical explanation: The turbulence intensity in the ice cavity should be very weak, as there is no wind forcing and presumably weak tidal currents. Thus, it is reasonable to assume that the buoyant layer of the freezing-point water in contact with ice shelf is fairly shallow, having a depth that is small compared with the height of the openings in the LR, typically about 800 m. The widths of the gaps in the ridge are also generally wider than the internal Rossby radius, which should be less than 10 km. In this regime, the outflow in the ridge gaps will be subjected to rotational hydraulic control and a rough upper bound on the volume transport is given by ref. 48


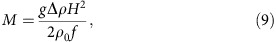


where *g* is the acceleration of gravity, *f* the Coriolis parameter and *ρ*_0_=1,000 kg m^−3^ a constant reference density. The diapycnal upwelling into the upper layer in the ice cavity is equal to *M*_A_, the inflow of water from the outside. We use a simple but physically well-founded representation of *M*_A_


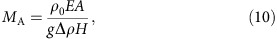


where *E* is the supply of mixing energy per unit area. Further, when *Q* is negligible,

Equation (4) implies that *F/M* ≈ *c*Δ*T/L* << 1. Thus, to a good approximation *M=M*_A_, which yields the following steady-state relations for the upper-layer depth and volume flow


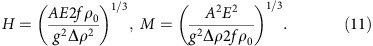


**Figure 7:**
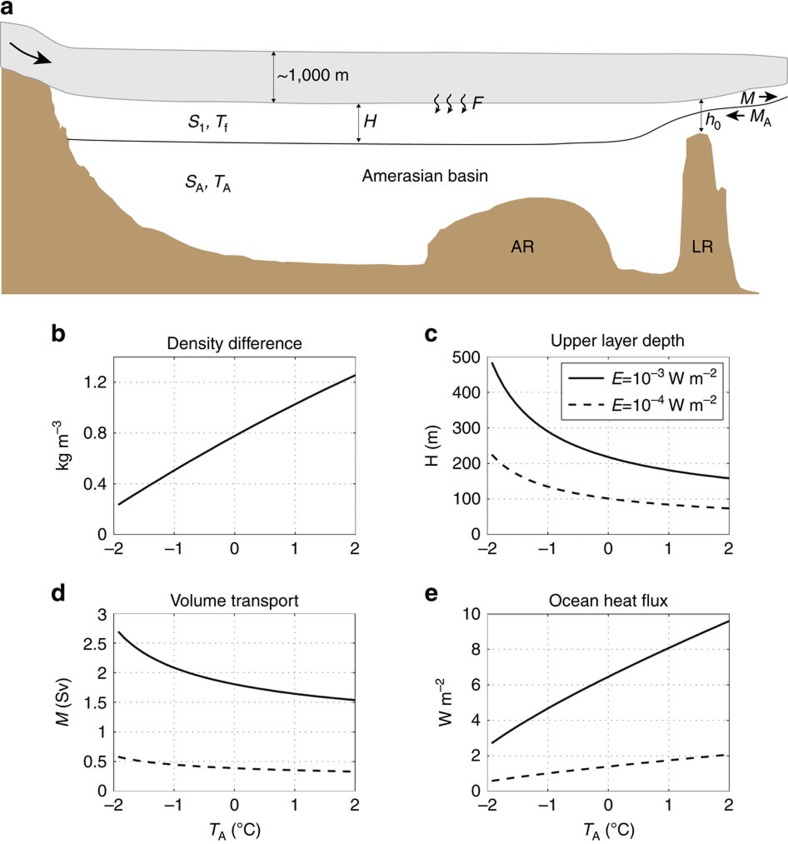
Basic components of the conceptual oceanographic model developed for the ‘ice cavity' in the Amerasian Basin underneath an ice shelf and calculated features of the cavity flow. (**a**) The ice shelf oceanographic model is assuming that the Lomonosov Ridge (LR) act as a barrier with a few open passages (*h*_0_) for water exchange to the Eurasian Basin, in turn connected to the World Ocean through the 2,500 m deep Fram Strait. (**b**–**e**) Calculated features of the cavity flow beneath an ∼1,000 m thick ice shelf as a function of the ambient ocean temperature *T*_A_; the minimum value of *T*_A_ is set to the surface freezing temperature for *S*_A_=35, which is about −2 °C. The oceanic heat flux is computed as *c*Δ*TM*/*A*, where *A*=3 × 1,012 m^2^ is the approximate area of the Amerasian Basin. A heat flux per unit area of 1 W m^−2^ corresponds to a basal ice-shelf melt of about 0.1 m per year.

Here, we assume that these relations provide rough upper bounds on the cavity upper layer depth and the exchange flow for given values of the ambient water temperature *T*_A_ and the mixing energy supply *E*. Figure 7 shows how the density difference, upper-layer depth, volume flow and oceanic heat transports vary with the temperature of the water outside the ice cavity. We have no information on the diapycnal mixing intensity. Therefore, the flow features are calculated for two values of the mixing energy input: *E*=10^−3^ W m^−2^, a value representative for the present-day Arctic Ocean, and *E*=10^−4^ W m^−2^. Given the absence of wind-generated turbulence in the cavity, the former value is certainly too large. Focusing on the more feasible case with *E*=10^−4^ W m^−2^, one finds that the results are not strongly sensitive to the ambient water temperature.

## Additional information

**How to cite this article:** Jakobsson, M. *et al*. Evidence for an ice shelf covering the central Arctic Ocean during the penultimate glaciation. *Nat. Commun.* 7:10365 doi: 10.1038/ncomms10365 (2016).

## Supplementary Material

Supplementary Information Supplementary Figures 1-3
