# Supplementary material for: Evidence for an ice shelf covering the central Arctic Ocean during the penultimate glaciation
Source: Nat Commun. 2016 Jan 18;7:10365. doi: 10.1038/ncomms10365 (PMC4735638; doi:10.1038/ncomms10365)
Supplement: Supplementary Information — Supplementary Figures 1-3 [file ncomms10365-s1.pdf]

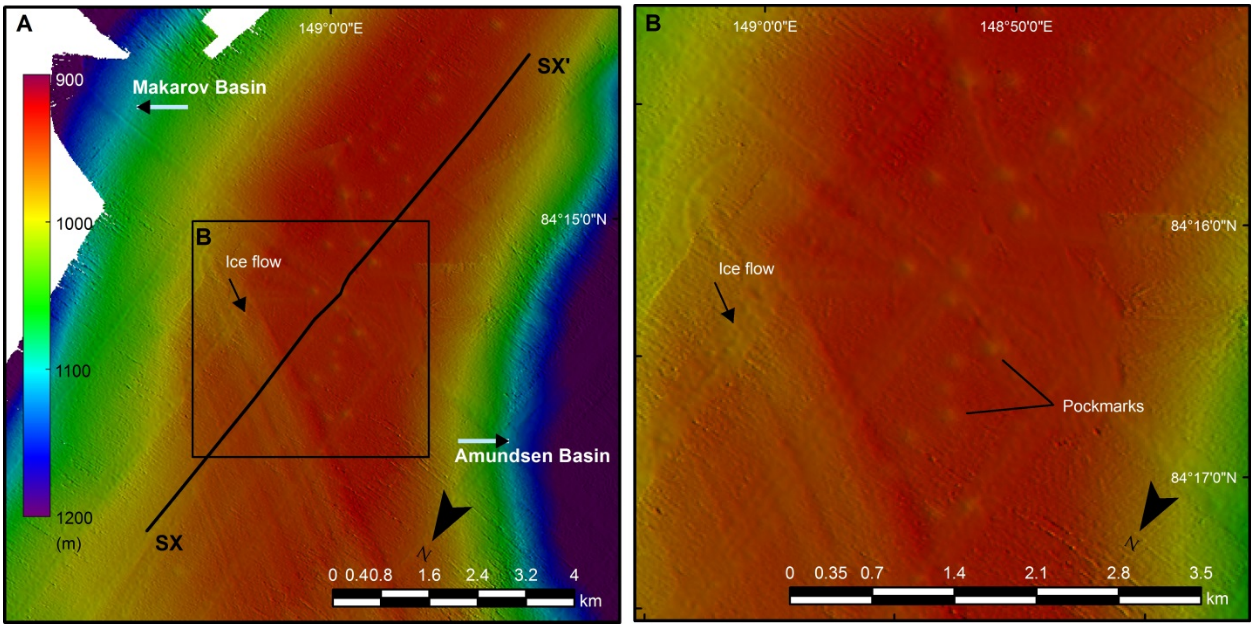

Supplemntary Figure 1. Multibeam imagery of pockmarks from the central Lomonosov Ridge next to the ice grounded area. The location of the sub-bottom profile shown in Supplementary Figure 2 is marked by the black line between SX – SX'.

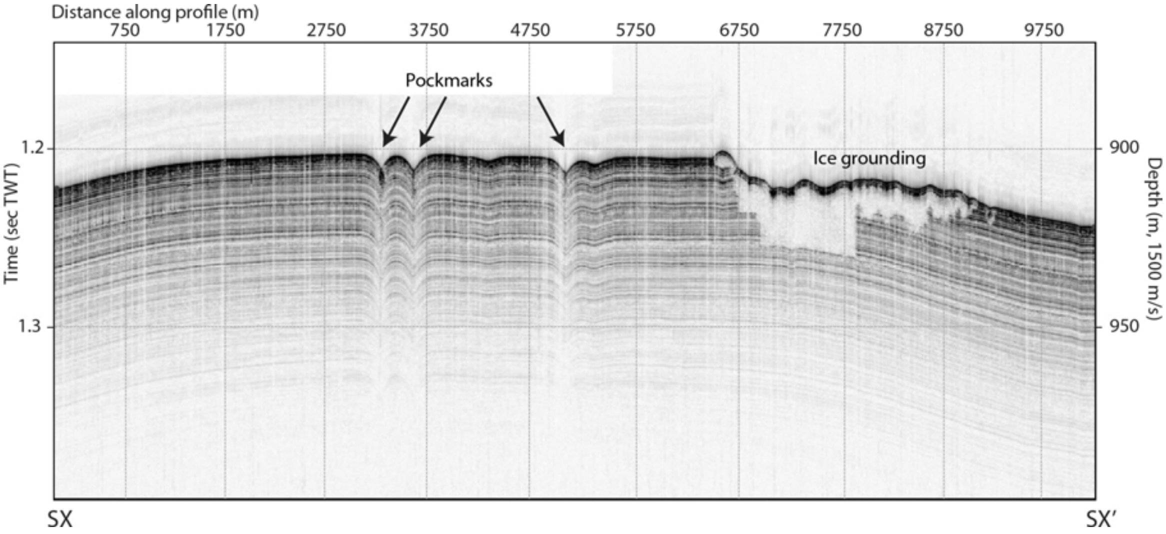

Supplementary Figure 2. Chirp sonar sub-bottom profile between SX and SX' through pockmarks. The profile location is shown on the map in Supplementary Figure 1.

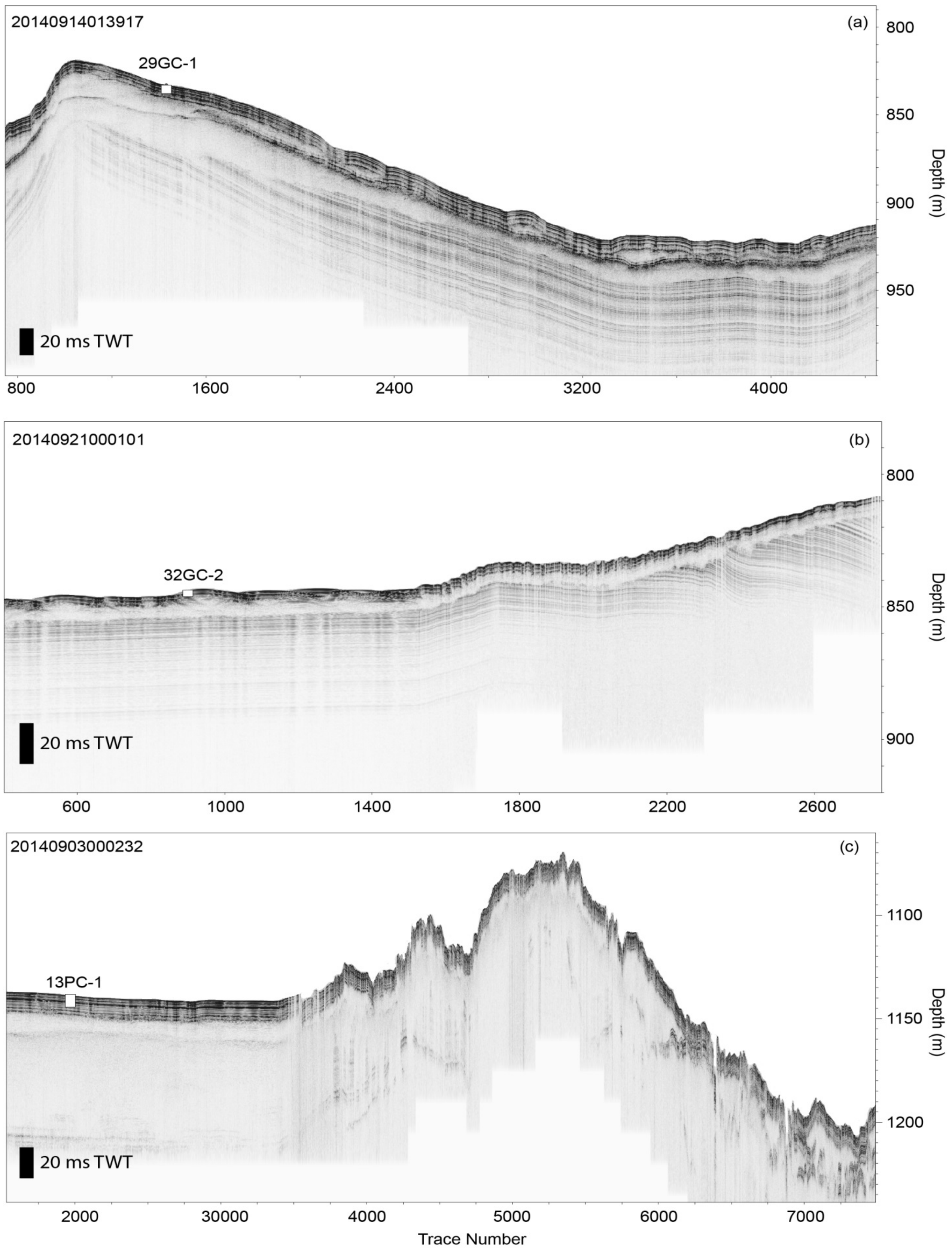

20140914013917
(a)
29GC-1
20 ms TWT
800
850
900
950
Depth (m)
800
1600
2400
3200
4000
20140921000101
(b)
32GC-2
20 ms TWT
800
850
900
Depth (m)
600
1000
1400
1800
2200
2600
20140903000232
(c)
13PC-1
20 ms TWT
1100
1150
1200
Depth (m)
2000
30000
4000
5000
6000
7000
Trace Number

Supplementary Figure 3. Chirp sonar sub-bottom profiles across key coring sites. All cores penetrated undisturbed acoustically stratified sediments deposited after the latest ice scouring event. (a) The 4.66 m long SWERUS-L2-13-PC1, on the Southern Lomonosov Ridge, (b) The 2.5 m SWERUS-L2-32-GC2 from the Lomonosov Ridge at 85°N and 153°E, and (c) The 6.14 m SWERUS-L2-13-PC1 from the Arlis Plateau. Depths for the sub-bottom profiles were calculated assuming at sound wave velocity through the water column and sediments of 1500 m/s.
